# Innovative Systems for the Delivery of Naturally Occurring Antimicrobial Volatiles in Active Food-Packaging Technologies for Fresh and Minimally Processed Produce: Stimuli-Responsive Materials

**DOI:** 10.3390/foods13060856

**Published:** 2024-03-11

**Authors:** Patricia Esteve-Redondo, Raquel Heras-Mozos, Ernest Simó-Ramírez, Gracia López-Carballo, Carol López-de-Dicastillo, Rafael Gavara, Pilar Hernández-Muñoz

**Affiliations:** Packaging Group, Institute of Agrochemistry and Food Technology (IATA-CSIC), Av. Agustín Escardino, 7, 46980 Paterna, Spain; pesteve@iata.csic.es (P.E.-R.); r.heras@iata.csic.es (R.H.-M.); esimo@iata.csic.es (E.S.-R.); glopez@iata.csic.es (G.L.-C.); clopezdedicastillo@iata.csic.es (C.L.-d.-D.); rgavara@iata.csic.es (R.G.)

**Keywords:** volatile antimicrobial compounds, fresh and minimally processed produce, active packaging, delivery systems, reversible covalent chemistry, stimuli-responsive materials

## Abstract

Certain naturally occurring volatile organic compounds are able to mitigate food spoilage caused by microbial growth. Their considerable vapor pressure enables them to create an antimicrobial atmosphere within a package, and this property can be used for the development of active food-packaging technologies. The volatility of these molecules, however, makes their stabilization difficult and limits their effectiveness. Whilst much research is being undertaken on the use of natural antimicrobial volatiles for inhibiting microbial growth in food, less attention has been paid to the design of controlled-release mechanisms that permit the efficient application of these compounds. Most studies to date either spray the volatile directly onto the fresh product, immerse it in a solution containing the volatile, or embed the volatile in a paper disc to create a vapor in the headspace of a package. More sophisticated alternatives would be delivery systems for the sustained release of volatiles into the package headspace. Such systems are based on the encapsulation of a volatile in organic or inorganic matrices (cyclodextrins, electrospun non-wovens, polymer films, micelles, molecular frameworks, etc.). However, most of these devices lack an efficient triggering mechanism for the release of the volatile; most are activated by humidity. All of these techniques are revised in the present work, and the most recent and innovative methods for entrapping and releasing volatiles based on reversible covalent bonds are also discussed.

## 1. Introduction

Improving food safety and shelf life continues to be of great interest to the food industry and cutting-edge science. Nowadays, huge efforts are being made to develop more sustainable food packaging and more efficient packaging technologies to ensure food quality and safety, extending the shelf life of products, and subsequently reducing food waste. In this context, the concept of active packaging technology arises based on packaging systems that are designed to emit or capture substances from the food or its environment to delay product deterioration [1]. Active packaging technologies offer an additional level of protection beyond traditional packaging methods. These systems provide diverse functionalities, which depend on their application (moisture or oxygen absorbers, ethylene or carbon dioxide regulators, absorbers of undesired compounds, or releasers of antimicrobial or antioxidant compounds) [2]. In particular, antimicrobial food packaging (AFP) can be highlighted since it can provide innovative solutions to extend the shelf life of food, by inhibiting or retarding microbial growth on foods via the action of antimicrobial compounds incorporated into the packaging system. The addition of these bioactive molecules to the polymeric matrix that constitutes the package walls present certain advantages in comparison to their direct incorporation into the food, as it can promote a controlled release of the antimicrobial compound at the food surface over storage, where it is usually needed. Additionally, the bioactive released from the polymer matrix minimizes the required doses of preservatives on food matrix, reducing possible molecular interactions between the active compound and the food matrix, which could cause a loss of its activity [3].

Although there are many types of antimicrobials, their effectiveness depends on several factors, including the method of incorporation, their mode of action, and the packaged food product. Thus, volatile and non-volatile antimicrobial compounds have been employed as antimicrobial agents for active food packaging. Particularly, polymeric materials with non-volatile compounds require direct contact with the food product (active polymer/food system) and include organic acids [4], peptides [5], enzymes [6] microorganisms such as yeasts or lactic bacteria [7], bacteriophages [8], Lauric arginate ethyl ester (LAE) [9], cationic polymers [10], and nanosized metal oxides [11,12]. Meanwhile, materials with volatile bioactive compounds can be easily released into the headspace as vapor or gas to inhibit microbial growth (active polymer/headspace/food system); direct contact with the food is not required, making them far more versatile and interesting for AFP design.

Antimicrobial natural volatiles comprise a large variety of molecules such as alcohols, aldehydes, and terpenes, among others, which are usually present in plants. These compounds are small molecules that exhibit certain volatility, and present efficient biological activities, such as antimicrobial activity, among others [13,14], rendering them highly suitable for use as natural preservatives. When these molecules are incorporated into packaging materials, they can be released over time, creating a protective atmosphere for the packaged food. However, they often exhibit a pungent odor, and their physicochemical properties, coupled with their inherent volatility, can complicate their incorporation into a polymer matrix through conventional converting procedures. Thus, losses during film processing or during its storage could reduce the content of the volatile molecules, compromising and even exhausting their antimicrobial activity [15,16,17]. Therefore, the incorporation of volatiles into active packaging design requires (a) innovative technologies to stabilize them during storage; and (b) a mechanism to control their release to ensure their effectiveness when needed [18]. In this line, some studies have focused on the use of novel structures to promote stabilization of volatiles as cyclodextrins, nanoclays, or zeolites [19]. However, these active devices do not release the volatiles in a precise manner. Their release is usually triggered by the moisture accumulated inside of the package, which causes the plasticization of the polymer, aiding the diffusion of bioactive molecules through the polymer. In order to improve this system, a new approach is emerging for the smart delivery of antimicrobials, namely using reversible covalent bonds of bioactive compounds. Reversible covalent bonds enable the active compound to be covalently anchored to the polymer matrix and to be released under specific conditions [20]. This technique does not present some of the limitations of the other systems, allowing for greater loading efficiency and better solubilization of the active molecule in the polymer, a high volatile stabilization, and a great control over its release [21]. However, although this technology has been used in a several fields such as cosmetic, pharmaceuticals, drug delivery, and agriculture [22,23], its use in AFP is scarce [24]. 

From these insights, the importance of a comprehensive review of innovative antimicrobial packaging solutions based on polymers that incorporate volatiles with biological activity is highlighted. In particular, the aim of this review is to discuss novel technologies for the incorporation of antimicrobial volatiles into polymers, which minimize volatile losses and improve their control release. The focus will be on the different types of delivery systems for volatile compounds, emphasizing the reversible covalent bonds of these molecules, which are released upon external stimulus, creating stimuli-responsive materials for active food packaging. Furthermore, this review explores existing applications of the reversible covalent bond approach in active food packaging, and the challenges that this technology addresses for its efficient application.

## 2. Antimicrobial Volatile Compounds in Food Packaging

Incorporating a bioactive volatile into the polymeric matrix can enhance its biological activity and confer new functionalities to the polymer. Antimicrobial packages containing bioactive volatiles are designed to release the volatile compounds in the package headspace where they exert their function, with those commonly employed being chlorine dioxide, plant extracts, and essential oils (EOs). In particular, the most extensive and recent research in this area has focused on the application of EOs and/or their active compounds.

EOs are composed of organic molecules extracted from various parts of plants, fruits, or spices. EOs have been extensively researched in the field of food packaging and include those extracted from jasmine, rosemary, peppermint, cinnamon, oregano, thyme, cumin, eucalyptus, rosewood, clove, tea tree, lavender, lemongrass, bergamot, or lemon [25], among others. Their chemical composition is highly variable and depends on various factors such as the plant part, genetics, climatic and geographical factors, seasonal conditions, harvesting time, or extraction techniques. Esters, ketones, acids, amines, alcohols, and aldehydes are among the diverse molecules present in EOs. They are generally lipophilic, highly volatile, and possess a distinct aroma. These substances are recognized for their remarkable antimicrobial and antioxidant properties [25]. Moreover, in the food industry, they are widely used as flavor enhancers, which are acknowledged for their safety, with FDA approval as Generally Recognized as Safe (GRAS) compounds. Only the volatile compounds that are authorized and considered safe for human consumption can be used as food additives, and therefore as bioactive compounds to be incorporated in polymer matrix, as there is a possibility of their migration into the packaged food. Consequently, EOs emerge as crucial additives in polymeric materials for the development of effective and consumer-demanded antimicrobial active food packaging. 

Several investigations have showcased the potential advantages of incorporating EOs and their main compounds into packaging as antimicrobial agents, while also maintaining the inherent qualities of the product. For instance, clove, thyme, and cinnamon oils serving as antibacterial agents, have been applied in meat to hinder the proliferation of foodborne pathogens, including *Staphylococcus aureus* (*S. aureus*), *Salmonella typhimurium* (*S. typhimurium*), *Escherichia coli* (*E. coli*), and *Listeria monocytogenes* (*L. monocytogenes*) [26,27,28]. In terms of antifungal properties, essential oils such as lavender, red thyme, clove, or vanillin have been reported to effectively inhibit the growth of *Botrytis cinerea* (*B. cinerea*) on both strawberries and grapes [29,30]. In this line, the use of the main components of EOs instead of the whole extract is also widespread, such as cinnamaldehyde from cinnamon oil, citral from lemongrass oil, eugenol from clove oil, or thymol from thyme oil. These compounds exhibit excellent antimicrobial activity, mainly due to their lipophilic characteristics; they have the ability to engage with microbial cell membranes and mitochondrial lipids, inducing structural changes and enhancing permeability [31,32,33].

In this review, we thoroughly revised antimicrobial food-packaging systems based on the incorporation of two types of volatile compounds: terpenes and aldehydes. Table 1 shows an example of the main antimicrobial compounds and their use in food packaging to inhibit or delay antimicrobial growth. 

### 2.1. Terpenes

Terpenes are organic compounds found in extracts from various plants, such as eugenol, thymol, carvacrol, linalool, and d-limonene, substances that have gained relevance for their role in food preservation. These compounds exhibit bioactive properties, including antimicrobial and antioxidant capabilities, making them valuable agents for enhancing the shelf life of food products. Experiments in vitro involving carvacrol, eugenol, carvone, and thymol, among other terpenes, have demonstrated their antibacterial effectiveness against *E. coli* and *S. aureus* [53], with eugenol and thymol being the most effective ones. In the context of food preservation, various terpenes have been used, individually or in combination, to extend the shelf life of various types of foods by inhibiting the growth of various microorganisms, as is shown in Table 1. Eugenol (4-allyl-2-methoxyphenol, C_10_H_12_O_2_) exhibits excellent antimicrobial activity in vitro against several food pathogens such as *S. aureus* and *E. coli* [54]. Its antibacterial activity suggests possible applications in meat preservation [34]. Additionally, eugenol has demonstrated antifungal properties, contributing to the prolonged freshness of strawberries [35]. Thymol (5-methyl-2-(propan-2-yl) phenol, C_10_H_14_O) is another noteworthy terpene with antibacterial effectiveness with potential in the field of meat products [36,37]. Its antifungal activity has also been shown against *B. cinerea*, a known mold causing fruit spoilage in tomatoes [38]. Extensively researched alongside carvacrol (2-methyl-5-(propan-2-yl) phenol, C_10_H_14_O), these two compounds have been combined to enhance the shelf life of strawberries [39]. Other terpenes such as linalool (3,7-dimethyl-1,6-octadien-3-ol, C_10_H_18_O) and d-limonene (1-methyl-4-(prop-1-en-2-yl) cyclohex-1-ene, C_10_H_16_) have also shown antibacterial activity against *E. coli*, *Salmonella enterica* (*S. enterica*), *S. aureus*, and *L. monocytogenes*, being more effective against Gram-negative bacteria [55]. Linalool has been applied in food and shown to be effective in preventing the growth of *L. monocytogenes* in fresh chicken breast during low-temperature storage [40]. In addition, studies on blueberries treated with d-limonene and liposomes reduced blueberry loss during storage by one third at the end of nine weeks [41].

Collectively, these terpenes showcase their potential in enhancing food preservation, offering natural and effective alternatives for extending the shelf life of various food products while maintaining their quality and safety.

### 2.2. Aldehydes

Natural aldehydes are also found in essential oils and plant extracts. These are low-molecular-weight compounds that are highly volatile. They exhibit certain biological properties, with their antimicrobial activity against fungi and bacteria being particularly noteworthy [56,57]. Table 1 also lists aldehydes that have been recently used to extend the shelf life of diverse foods.

Trans-2-hexenal ((E)-hex-2-enal, C_6_H_10_O) is an α, β-unsaturated aldehyde classified as a green-leaf volatile due to its association with the characteristic green color found in plants. In food applications, this compound has been efficiently proven to be an antifungal agent able to reduce common fungal spoilage in pears, kiwifruit, blackberries, and apples [42,43,44,45]. In addition, this aldehyde was found to be effective against other microorganisms responsible for fruit spoilage in minimally processed fresh produce such as fresh-cut pineapple [46]. The mechanism behind this antifungal action involves α, β-unsaturated aldehydes penetrating cells and reacting with biologically important nucleophilic groups [58].

Other α, β-unsaturated aldehydes such as cinnamaldehyde ((2E)-3-phenylprop-2-enal, C_9_H_8_O) and citral ((2E)-3,7-dimethylocta-2,6-dienal, C_10_H_16_O) have been applied as antimicrobial agents. Cinnamaldehyde was used in the form of an emulsion to significantly improve the quality of mushrooms, acting by decreasing respiration rate, weight loss, and *Pseudomonas* count and improving firmness and color [47]. In fresh fish, Hosseini et al. [48] developed chitosan nanoparticles loaded with cinnamaldehyde and embedded in ternary chitosan/poly(vinyl alcohol)/fish gelatin matrices to extend the shelf life of rainbow trout fillets to 12 days. On the other hand, citral has been widely studied for its antimicrobial activity and has also been applied in foods, for example as an antimicrobial in a salad, being effective against enterobacteria, lactic acid, psychrotrophic bacteria, yeasts, and molds [49]. Other aldehydes, such as salicylaldehyde and vanillin, have also been studied in active food packaging. Salicylaldehyde (2-hydroxybenzaldehyde, C_7_H_6_O_2_) has been used to inhibit natural microbial load growth in fresh-cut pineapple [46] and to inhibit *E. coli* growth in orange and carrot juice [51]. The antimicrobial properties of vanillin (4-hydroxy-3-methoxybenzaldehyde, C_8_H_8_O_3_) were also applied to improve the quality of fresh-cut melon, which was stored for 10 days at 5 °C: the results showed a significant antimicrobial effect against mesophilic bacteria, with a reduction of 1.5 log colony forming units (CFU)/g and a remarkable decrease in Enterobacteriaceae by 2.2 log CFU/g [52]. 

In summary, these studies underscore the antimicrobial potential of diverse volatile molecules as natural and effective agents for inhibiting microbial growth and preserving the freshness and quality of various food products, as well as their implementation in active food packaging. 

### 2.3. Methods for the Incorporation of Antimicrobial Volatile Compounds in Polymer Materials

In the context of antimicrobial active packaging, there are many studies that spray the volatile directly onto the fresh product, immerse it in a solution containing the volatile, or embed the volatile in a paper disc to create a vapor in the headspace of a package. In recent years, several technologies have been developed to incorporate volatiles into polymeric matrices, such as new encapsulation technologies or nanotechnology. However, very few studies have applied these materials to real food matrices or packaging. The main advantage of incorporating these active compounds in polymeric material is the controlled release of the volatile on the food surface, reducing the amount of antimicrobial preservatives needed, as it acts on the food surface where most microbial spoilage occurs. Moreover, their incorporation into polymers allows high stabilization and less degradation. Table 1 summarizes examples of different methods of incorporating volatiles and their application in food, along with the main microbial targets.

#### 2.3.1. Direct Incorporation into the Polymeric Matrix

There are several methods of incorporating active volatiles directly into polymer matrices for food packaging, each with pros and cons.

Solvent casting has been the most widely used method, easily implemented in a laboratory setting, noted for its cost-effectiveness and the use of low processing temperatures. However, it has inherent drawbacks, such as the rapid migration of volatile compounds, which causes a great organoleptic impact on the food product at short durations of exposure. In addition, this method is characterized by a slow process due to the necessary drying phase (especially slow in aqueous formulations), during which evaporation of volatile compounds can occur, with consequent losses. For example, Martínez-Abad et al. [59] incorporated cinnamaldehyde into polycaprolactone (PCL) films through solvent casting. They reported that approximately 71% of the active compound was lost in the films during solvent evaporation. Although, in a sealed environment, a concentration of 5.34 mg of cinnamaldehyde per L of air was released from PCL films containing 20% (*w*/*w*) of cinnamaldehyde, which caused a complete inhibition of bacterial growth of *S. enterica* and *L. monocytogenes* at 4 °C and 10 °C for at least 30 days. Sodium alginate films containing cinnamon essential oil and citric acid as volatile and non-volatile antimicrobial agents, respectively, were also obtained through the casting method [60]. The antimicrobial efficacy of these films was evaluated against *Listeria innocua* (*L. innocua*) and *E. coli*. In a disc diffusion assay, they found antimicrobial activity in both bacteria. However, in a vapor diffusion test, films containing EOs were evaluated, since there is no direct contact with bacteria. After 6 days of contact, the growth of *L. innocua* and *E. coli* was reduced growth by around 5 log CFU/filter. The addition of two types of antimicrobial agents, volatile and non-volatile, allows for the broadening of their use. Lee et al. [60] assessed the antimicrobial potential of films on sliced cooked ham inoculated with *L. innocua*. Although their in vitro test showed great inhibition, the results on food showed a lack of effectiveness of the volatile EO, whereas films containing citric acid fully inhibited the bacterial growth. This highlights the need for in vitro studies with real food, since volatile release could be influenced by changes in the diffusion rate under food storage conditions and interaction with the food matrix, which would negatively impact the antimicrobial activity.

Another commonly employed technique is extrusion. This method has gained prominence in the packaging industry due to its compatibility with industrial machinery. Despite its popularity, this technique has its own set of challenges. The high temperatures commonly employed during processing, exceeding 145 °C, may lead to volatilization or degradation of the volatile agents. Furthermore, introducing the volatile compound in the final stages of the compounding process, as a preventive measure against evaporation, can result in uneven distribution within the polymer matrix [19]. There are numerous studies that have investigated the addition of essential oils using this technique to inhibit antimicrobial activity [61]. For example, bakery products were packaged on thermoformed trays of PLA obtained through cast sheet extrusion containing carvacrol and cinnamaldehyde, and mold spoilage was delayed for 2 days in trays with 8% (*w*/*w*) of cinnamaldehyde and carvacrol, compared to the control [62]. 

Another alternative is the coating of conventional materials using printing methods, although there may also be adhesion compatibility problems with the polymeric matrices acting as substrates. For example, a study by Muriel-Galet et al. [49] involved the development of active polypropylene (PP) films coated with ethylene vinyl alcohol (EVOH) containing citral to enhance the shelf life of a salad. The packaging successfully achieved the inhibition of the growth of enterobacteria, lactic acid bacteria, psychrotrophic bacteria, as well as yeasts and molds at the beginning of storage. The coating technique has also been explored in the development of active coatings on food, employing biopolymers such as pectin [63], chitosan [64], starch [65], and alginate [66], among others. Polymer coatings including volatile compounds have also been developed to extend the shelf life of strawberries [67] and tomatoes [68].

Finally, supercritical CO_2_ impregnation is another alternative method for incorporating active additives into polymer matrices, offering a non-toxic and safe approach. This technique utilizes chemically inert CO_2_ as a solvent, allowing for the impregnation of various natural and synthetic polymers with high diffusion coefficients and solvent capacity at relatively low temperatures. There are several advantages to this impregnation process. Firstly, it accommodates a wide range of natural and synthetic polymers that swell upon contact with supercritical carbon dioxide. Secondly, it is very effective at impregnating hydrophobic molecules such as essential oils. In addition, this method is able to work under mild and oxygen-free conditions, making it suitable for the impregnation of biologically active natural compounds. In one specific application, cinnamaldehyde was incorporated into poly(lactic acid) (PLA) films using supercritical carbon dioxide. The impregnated PLA films exhibited potent antibacterial activity against *E. coli* and *S. aureus* [69]. In summary, supercritical CO_2_ impregnation is a promising method for efficiently incorporating active compounds into polymer matrices. However, the initial investment cost associated with applying pressures ranging from 10 to 30 MPa, and the up-scaling, are major drawbacks. 

#### 2.3.2. Encapsulation

As mentioned above, the use of volatile compounds as food preservatives is hampered by their inherent volatility and their thermal instability, and requires encapsulation for long release processes. To overcome this challenge and reduce losses, several encapsulation methods have been investigated. These efforts are aimed not only at improving solubility and compatibility with polymer matrices, but also at reducing the degradation of volatile compounds during processing, packaging, manufacturing, or storage. In addition, efforts have been made to minimize the organoleptic impact on food products due to the strong odors associated with essential oils [70].

Encapsulation methods encompass a range of approaches, including physical techniques such as spray drying, spray chilling, spray coating, layer-by-layer, electrospinning, extrusion, centrifugation, fluidized bed, supercritical fluids, co-crystallization, and lyophilization. Physicochemical methods involve simple or complex coacervation, micelles, liposomes, emulsions, lipid matrices, and solvent evaporation. Chemical methods, on the other hand, comprise interfacial polymerization and molecular inclusion [71]. Choosing the optimal encapsulation technique for gaseous/volatile active compounds requires consideration of specific physicochemical and molecular requirements. One intriguing procedure in this field is the application of supramolecular chemistry, which involves incorporating small molecules within structural cavities associated with intermolecular forces.

Layer-by-layer self-assembly (LBL) is a technique that employs several polymers with opposite charges, which are deposited alternatively to form a film/coating. For instance, an edible multilayer coating was developed with chitosan and pectin to enhance the shelf life of strawberries [72]. Moreover, bioactives can be incorporated into polymeric matrices to enhance their antimicrobial activity. For example, lemon essential oil and ε-polylysine were added to chitosan and carboxymethyl cellulose to form a multilayer material, and the volatile retention was higher on bilayer films than those with the chitosan–lemon essential oil, showing a reduction in their diffusion rate and losses caused by the volatility [73]. The effectiveness of these films was tested on apples, pears, and peaches. The fruits treated with active multilayer material containing 1.5% (*w*/*v*) of lemon essential oil showed an inhibitory effect on fungal incidence compared to the control. Polymer concentrations between 0.5 and 3% (*w*/*v*) were evaluated. The antimicrobial effectiveness improved by increasing the polymer content; however, the authors reported that using polymer concentrations above 1.5% (*w*/*v*) resulted in films with bad properties [73]. LBL has been also employed to control the release of bioactives; thus, thyme essential-oil microcapsules were developed combining chitosan and sodium alginate with several different amounts of layers (0, 2, 4, and 6). These authors showed that release of the bioactives could be controlled by the storage temperature and number of layers, as well as by changes in the pH [74]. The material showed antibacterial activity against *S. aureus*, *E. coli*, and *Bacillus subtilis* (*B. subtilis*) in an in vitro test. In fact, in milk, the presence of microcapsules with thyme essential oils resulted in a significant reduction in *S. aureus.*

Electrospinning has been thoroughly explored to encapsulate bioactive compounds. Electrospinning is an economical method for fabricating fibers from a variety of natural and synthetic polymers. In this line, Altan et al. [75] developed fibrous composite films from zein and PLA incorporating carvacrol at three different concentrations (5, 10, and 20%) using electrospinning. Their results showed successful encapsulation of carvacrol, resulting in improved sustained release. Films composed of fibrous zein and PLA containing 20% carvacrol inhibited mold and yeast growth by 99.6% and 91.3%, respectively. 

In addition to this widely used technique, the latest research in encapsulation of volatiles for food packaging focuses on the use of nanocarriers, such as nanoclays, nanoparticles, or cyclodextrins. 

**Figure 1:**
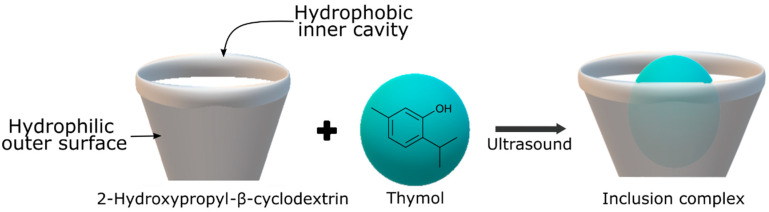
Inclusion complex process between thymol and 2-hydroxypropyl-β-cyclodextrin.

Cyclodextrins, cyclic oligosaccharides derived from starch molecules through enzymatic modification, exhibit a unique capability in encapsulating hydrophobic molecules within their hydrophobic central cavities. This results in the formation of inclusion complexes, altering the physical, chemical, and biological properties of the encapsulated guest molecules. Acting as “empty capsules” with various molecular sizes, cyclodextrins enhance shelf stability and facilitate material handling. Their advantages include non-toxicity, biodegradability, and biocompatibility, contributing to their Generally Recognized as Safe (GRAS) status. Within the range of inclusion complexes used in food packaging, the incorporation of antimicrobial agents is the most extensively researched. Sun et al. [38] synthesized a thymol inclusion complex with 2-hydroxypropyl-β-cyclodextrin using ultrasonic technology, as shown in Figure 1. This treatment showed a remarkable inhibition of *B. cinerea* in tomatoes, demonstrating the potential of ultrasound-assisted complexation to improve the solubility and stability of thymol as an antifungal agent in fruit preservation. Moreover, encapsulation of citral and trans-cinnamaldehyde in β-cyclodextrin was able to extend the shelf life of beef by about 4 days at 4 ± 1 °C [50].

**Figure 2:**
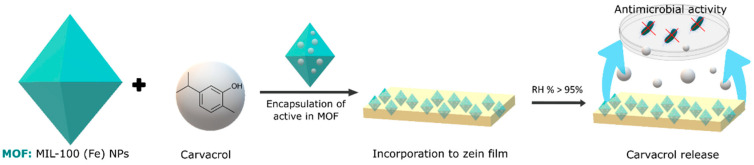
Representation of the encapsulation process of carvacrol in an MOF, in particular MIL-100 (Fe) nanoparticles and their incorporation in polymeric films to control the release of carvacrol and exert its antimicrobial activity.

Finally, metal–organic frameworks (MOFs) represent another category of materials with great encapsulation potential. These crystalline structures, composed of metal ions or clusters linked by organic bridging ligands, form network structures with one-, two-, or three-dimensional configurations. Unlike cyclodextrins, MOFs rely on pore size, porosity, and surface area for the adsorption of gases and volatiles. The specific structural properties of MOFs allow for precise control of the adsorption mechanisms, offering a unique perspective on encapsulation. These structures have not yet been widely used in food; however, there are several studies using them to encapsulate volatile antimicrobial compounds. Caamaño et al. [76] developed a material based on an organometallic framework (mesoporous iron(III) trimesate nanoMOF) that encapsulated a natural food preservative molecule, carvacrol, following a direct and biocompatible impregnation method as schematized in Figure 2. Prolonged activity against *E. coli* and *L. innocua* was achieved. In the case of *E. coli*, a reduction of 1.76 in colony forming units (CFU) was achieved for the carvacrol encapsulated with the MOF, and this was 1.57 CFU for *L. innocua*. This translates to an 82% increase in the antimicrobial effect of carvacrol for *E. coli* and a 93% increase for *L. innocua* following encapsulation. Wu et al. [77] used a zinc metal–organic framework as a vehicle for loading thymol. The resulting thymol–zinc metal–organic framework exhibited effective antibacterial activity against *E. coli* O157:H7 by achieving a 4.4 log reduction in 24 h due to the sustained release of thymol, demonstrating a potential application in food packaging. 

### 2.4. Release Mechanisms of Volatile Compounds from Non-Stimuli-Triggered Systems

In the design of packaging, the active compound can be embedded inside the film (encapsulated or not), or added in a pad or sachet, or deposited as a coating on the surface of the polymer layer. The release of volatile compounds in food active packaging is often achieved through monolithic administration systems. In these systems, the active molecule is uniformly dispersed within the polymeric material of the packaging, and the release occurs through the diffusion of the active agent along the polymeric layer into the surrounding environment. Although this system is widely used, it has the disadvantage of not having a specific release mechanism, resulting in loss or degradation of the volatile before its use with the food product.

The compatibility of the volatile compound with the polymer matrix and the ability to retain volatiles during processing, transport, and storage are the most important parameters in determining the efficacy of an antimicrobial packaging. Factors influencing the retention and release mechanisms of volatiles include the concentration and type of polymer, the chemical structure and concentration of the volatile compound, the addition of fillers, plasticizers, and emulsifiers, the drying conditions, the material thickness, the relative humidity, the temperature, and the pH.

The success of active antimicrobial packaging relies on achieving an equilibrium between the controlled release of the active compound and the microbial growth kinetics. If the release rate does not reach the minimum inhibitory concentration (MIC) required for specific microorganisms, or if it cannot be sustained throughout the shelf life of the food due to overly rapid release, effective microbial inhibition cannot be attained. Therefore, monitoring and controlling the release of volatile compounds from the packaging material to the food sample is crucial to ensure their functionality. For that, kinetic studies of volatile release are needed to understand its further application in food packaging. 

Specific migration tests can be employed to evaluate the diffusion of active compounds within polymeric matrices under specific time and temperature conditions, tailored to the nature of the packaged food and its usage and storage characteristics [78]. For instance, Cerisuelo et al. [79] modelled the release of carvacrol from an EVOH coating on a PP film, characterizing the kinetics and degree of mass transport of carvacrol as a function of relative humidity. The absorption of water by the EVOH coating acted as a trigger mechanism for the activity, since it is a hydrophilic polymer that can be plasticized at high humidity and promote a high-rate release of the volatile.

Alternatively, the release of volatile compounds can be controlled by adding substances such as plasticizers, nanoclays, natural fibers, and cyclodextrins. β-cyclodextrins (β-CDs) are particularly interesting due to their ability to encapsulate hydrophobic molecules within their hydrophobic central cavities (Section 2.3.2). Chen et al. [50] developed an EVOH film containing bioactive components encapsulated in β-cyclodextrin as an inclusion complex. They determined the release rate of active antimicrobial agents, measured the thermal stability of the inclusion complexes, and verified the preservative effect of the active packaging film through their application with real foods, specifically refrigerated beef shank. The results showed that the active compounds encapsulated in the β-cyclodextrin as an inclusion complex exhibited high thermal stability and a slow release rate, thereby extending the shelf life of the refrigerated beef to 4 days at 4 ± 1 °C.

## 3. Reversible Covalent Bonds for Active Food Packaging

As mentioned above, traditional methods to incorporate antimicrobial volatiles in polymers for active food packaging have several disadvantages including the percentage loss of effective volatile incorporation and the lack of a specific controlled release mechanism of the active compound. To solve this problem, the production of reversible covalent bonds (RCBs) has emerged as a new method of volatile incorporation that allows a robust volatile stabilization, minimizing volatile losses while guaranteeing a specific triggered release mechanism. RCBs derive from the concept of click chemistry, which is based on the formation of stable compounds of diverse functionalities with fast and effective organic synthesis processes with high yields and short reaction times under mild reaction conditions [80]. An RCB is a type of covalent bond that possesses specific response properties to specific stimuli. The use of reversible covalent bonds excels both in the creation of new supramolecular polymeric materials [81,82] and in the modification of existing polymers on its surface or along the polymeric chain [83]. RCBs represent an advance over common covalent bonds due to the fact that their reversible properties are acquired by the modified material [84,85], and the bond remains stable over time, until a specific stimulus is applied [81]. This reversibility is interesting in the field of active packaging since it allows the reversible immobilization of volatiles and bioactive compounds along the polymer chain, whose release and action can be controlled and triggered under specific conditions (pH, humidity, light, heat, etc.). Due to all of the advantages that RCBs present over non-reversible covalent bonds, interest in them has become widespread for the development of stimuli-responsive materials in diverse fields, such as biomedicine, cosmetics, and pharmaceutics [22]. In the following section, the most important types of reversible covalent bonds, their mechanism of formation and its reversibility, their application for the controlled release of volatiles, and their use for the design of stimuli-response structures in the AFP field will be discussed.

### 3.1. Types and Mechanisms of Formation–Hydrolysis of Reversible Bonds

There are many types of reversible covalent bonds based on different formation–hydrolysis mechanisms. Some of the most common new bonding techniques due to their easy synthesis include Diels–Alder reaction adducts, hemiacetal or cyclic acetal bonds, disulfide bonds, and Schiff base reactions, which encompass the formation of acylhydrazone, oxime, and imine bonds, respectively [86]. Their formation–hydrolysis mechanisms and their applications are summarized in Table 2.

The Diels–Alder (DA) reaction leads to the formation of cycloadducts with a double bond (-C=C-) on the cycle via 4 + 2 (four carbons + two carbons) cycloaddition of a diene (-C=C-C=C-) to a dienophile (-C=C-) (Table 2) [103]. Its formation and reversible paths are controlled by the temperature applied to the system [104]. When temperatures below 110 °C are applied to the system, the formation of the cycloadduct is favored. However, if the temperature increases above 110 °C, the reversible path (retro-Diels–Alder reaction) takes place, leading to the bond’s cleavage. In addition, the retro-Diels–Alder reaction has been reported to occur under neutral physiological conditions [88]. The Diels–Alder reaction is applied on the formation of thermo-responsive self-healing polymers and drug delivery systems [87,88,89]. In the latter case, Li et al. [87] reported synthetized self-healing pH-responsive pectin/chitosan hydrogels for the immobilization of fluorouracil via Diels–Alder bonds, whose release was favored at a neutral pH, rather than acidic, under physiological conditions. 

Reversible cyclic acetals and hemiacetals can be formed via condensation between non-sterically hindered carbonyl groups (-C=O), mostly aldehydes and ketones, with diols (-C-OH) [105]. The formation of the acetal takes place under the presence of acid catalysts and its reversibility can be triggered by an acidic pH, with both mechanisms being influenced by temperature. Acetals are mostly used as pH-responsive drug delivery systems [91]. Cao et al. [106] designed an acetal-containing hyperbranched amphiphilic micellar block copolymer for the pH-responsive delivery of doxorubicin (DOX). The micellar copolymer showed a great release of DOX when subjected to slightly acidic pH levels in contrast with its release under neutral conditions. 

Disulfide (R-S-S-R) bonds are formed through the reaction of two thiol (-SH) groups and can take place through oxidation of the thiols or through ionization in basic conditions. Their reversible mechanism can be triggered by reducing the bond or by increasing the temperature of the bond with heat or light [107]. Disulfide bonds are mostly applied for the formation of drug delivery systems [90,92], such as the paclitaxel–citronellol conjugates linked by disulfide bonds developed by Sun et al. [92].

Schiff bases are an important group of click chemistry reactions based on the formation of carbon–nitrogen (-C=N-) double bonds through condensation reactions between carbonyl (-C=O) groups (mostly aldehydes or ketones) and nucleophilic primary amino groups (-NH_2_). Within Schiff bases, three types of reversible covalent bonds stand out: acylhydrazone bonds, oxime bonds, and imine bonds [108,109]. Reversible acylhydrazone (-NH-N=C-) bonds are formed through polycondensation of the carbonyl group (-C=O) of an aldehyde or ketone, with a hydrazine group (-NH-NH_2_) under acidic conditions [108]. The reversibility of this reaction occurs under temperature changes and under acidic pH levels [110]. Mostly, acylhydrazone reversible bonds have also been used to develop drug delivery systems [93]. Thus, Su et al. [94] reported obtaining pH-responsive complex micelles with hydrazine bonds anchoring docetaxel (DTX). The delivery of DTX was successfully studied when the micelles were submerged into acidic media. Reversible oxime (O-N=CH-) bonds are formed through condensation of hydroxylamine (O-NH_2_) with carbonyl groups of aldehydes or ketones [109], and are more stable than their two counterparts (not so reversible under acidic pH conditions) [111]. Their reversibility can be catalyzed by energy, applying methods like temperature control or UV radiation [112]. For this reason, oxime bonds are mostly used for the design of self-healing materials [95] but their use to release agrochemical pesticides via the action of UV light has also been reported [97]. 

Imine (-C=N-) reversible bonds are formed through condensation of carbonyl groups of aldehydes or ketones with primary amines (-C-NH_2_) [113]. The mechanism of imine formation occurs in the presence of water and is favored by acidic pH levels. The reversibility of this bond also takes place in the presence of water and is also improved by acidic pH. Imines stand out over other RCBs due to their easy formation under mild temperature conditions, high conversion ratios, and high reactivity when exposed to acidic pH conditions [114,115], as well as them guaranteeing the system with magnificent stimuli-responsive, recycling, and self-repair properties [86,116,117]. For this reason, imines are the most used RCB for the immobilization and release of diverse compounds in many fields from drug delivery to agriculture [22,23]. For example, the slightly acidic environment of tumoral tissue has been employed as a specific trigger for the release of anchored drugs [84,98]. Additionally, other strategies for bioactive release have been explored through the production of organic acids by microorganisms. Thus, the release of antibiotics through the hydrolysis of Schiff base reactions has been reported [99]. In the cosmetics field, Tchakalova et al. [20] employed imines to stabilize fragrances, which were efficiently released when needed. In the agricultural field, the obtention of covalently anchored hexanal to chitosan film has been reported, with active properties when subjected to acidic aqueous solutions [100]. 

Because of their pH-responsive properties and their high reactivity in both formation and reversible mechanisms, the imines are the preferred and the most reported RCB system for capturing and releasing volatiles in diverse areas, including stimuli-responsive materials in the field of food packaging. To develop stimuli-responsive systems applicable in active food packaging, understanding the volatile release mechanism is needed, since it will affect the design of the system.

### 3.2. Volatile Release Mechanism of Triggered Systems

**Figure 3:**
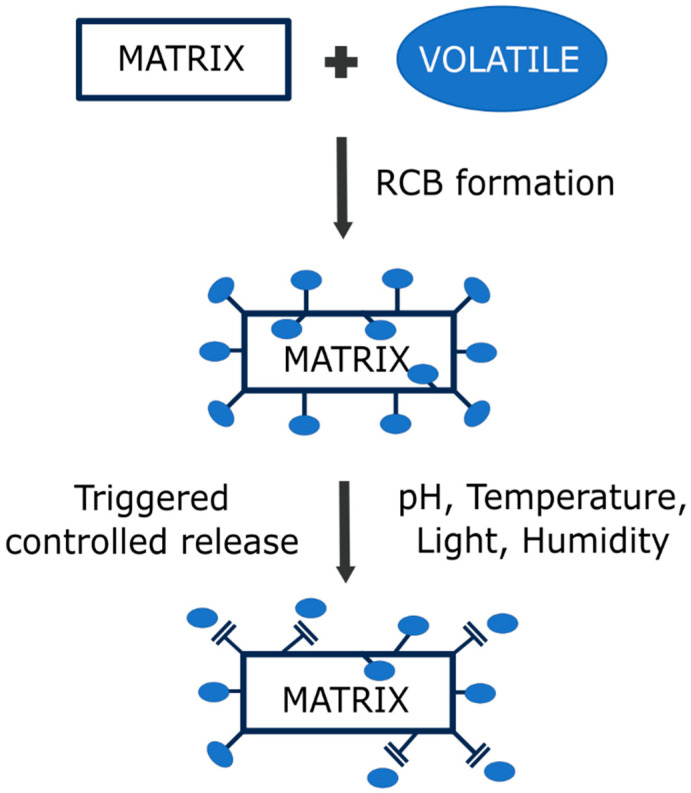
Concept of the formation of stimuli-responsive RCB-based materials and the triggering of volatile release when a stimulus is applied to the system.

As discussed above, the cleavage of reversible bonds can be triggered by different stimuli, such as humidity, pH, light, and temperature. Changing the pH conditions is the most common method of triggering the release of volatiles. Most volatiles used in active packaging applications are aldehydes, ketones, esters, and alcohols, which are stabilized by pH-sensitive imine reversible bonds. Despite the common uses of imines in the design of stimuli-responsive structures for the incorporation and release of volatile molecules, few works have studied their volatile release mechanisms [118]. Figure 3 explains the volatile release mechanism concept. 

The mechanism of volatile release from materials based on RCBs can be reduced to four steps: (1) the stimuli interacts with the matrix (e.g., water and acid in contact with the polymer); (2) cleavage of the bond occurs (hydrolysis of the bond); (3) the released compound diffuses to the surface of the polymer (if it is not already anchored on the surface); and (4) the volatile passes to the surrounding medium (water or air). In this sense, the porosity of the matrix, as well as the bonding point (surface or intern), influences the volatile release rate and its dynamics [119]. For instance, if the volatile compound is bonded to the surface of the polymer, the release mechanism will only be affected by the second and the fourth steps, and a high-porosity matrix (or a thermal labile matrix, in case other stimuli are applied) will not be needed. However, if it is bonded inside the polymer matrix, all steps must be taken into consideration, and a matrix compatible with the applied stimuli will be needed in order to improve the release rate. In this sense, several works have reported that imidazolidine precursors anchored to PLA, ethylcellulose (EC), and ethylcellulose-poly(ethylene oxide) (EC-PEO) fibers [120,121,122] exhibited a sudden but brief release of volatiles due to the anchorage of the aldehyde on the surface, requiring the fiber to swell in order to release the bonded aldehyde within. Also, the release rate will be dependent on the intensity or the concentration of the stimuli, the polymer matrix, the distribution of the bonds between the matrix and the volatile, and the dispersion of the volatile into the environment (which will be dependent on other factors like the nature of the volatile). In the same way, the quantity of anchored aldehyde molecules will change the release rate of the aldehyde. In most cases, the greater the number of anchored volatiles, the greater the release rate [118]. In addition to these considerations, it is important to remark that when using RCBs, there is always a thermodynamic equilibrium between the reagents and the products, and the release of the volatile will only occur when the material is exposed to stimuli. As soon as a stimulus is applied, the system (equilibrium) shifts towards a transition state; thus, the cleavage of the bond drives the immobilized volatile to transition from a normal state (solid within the matrix; product) to a final chaotic state (dissolved in water, gaseous in air; original reagent), which is the complete release of the volatile. Because of the thermodynamic equilibrium, the chaotic state will not be reached, causing a release of the volatile over time (controlled release). In conclusion, there is much to be studied about the mechanism and dynamics of volatile release in order to effectively apply the already-designed stimuli-responsive materials.

### 3.3. Design of Stimuli-Responsive Structures Using Reversible Covalent Bonds

In recent years, imines have been implemented in the design of stimuli-responsive materials for the release of volatile and bioactive compounds. Stimuli-responsive structures using imines for the immobilization and release of antimicrobial compounds are a revealing alternative to conventional systems controlled by diffusion in polymer matrices. These structures are nourished by the reversible character of imines, so that they can change their physical or chemical properties in response to pH stimulation [123]. To generate stimuli-responsive materials based on imine bonding, polymeric matrices with abundant amino groups are required. In this regard, chitosan is the most commonly used matrix for the design of stimuli-response structures with imine reversible bonds, followed by polyethyleneimine (PEI). Chitosan is a biodegradable biopolymer with amino groups distributed all along the polymeric chain, making it a suitable polymer for the incorporation of diverse bioactive compounds with an inherent antimicrobial capacity [124]. With a high barrier to gases but sensitivity to moisture, and poor thermoplastic properties, its application in the food industry, and specifically in active food packaging, has been reduced to its combination with other polymers [125]. It is an interesting polymeric matrix for the design of stimuli-responsive structures for the release of active compounds (mostly aldehydes) via imine bonds. 

**Figure 4:**
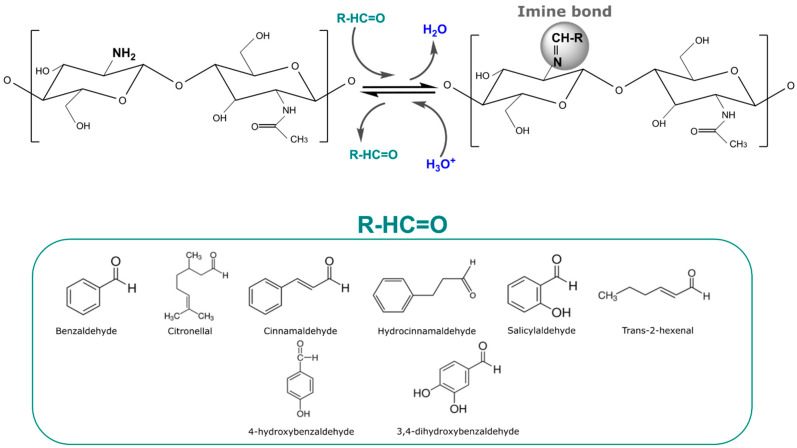
Scheme of the immobilization of aldehydes (benzaldehyde, citronellal, cinnamaldehyde, hydrocinnamaldehyde, salicylaldehyde, trans-2-hexenal, 4-hydroxybenzaldehyde, and 3,4-dihydroxybenzaldehyde) to chitosan films through imine reversible bonds and their release under acidic conditions.

The principal chitosan imine stimuli-responsive structures have been developed as films. These films can be prepared by casting directly from a solution with both chitosan and the volatile, or by previously preparing a chitosan film, and then carrying out its functionalization through a reaction of the volatile with the already-casted chitosan film [126]. Most works design stimuli-response chitosan films wherein the aldehyde release is activated by acidic pH conditions in aqueous solutions, subjecting the film to direct contact with the triggering factor [45,51,57]. In this sense, plenty of antimicrobial aldehydes such as benzaldehyde, citronellal, cinnamaldehyde, hydrocinnamaldehyde, salicylaldehyde, 4-hydroxybenzaldehyde, 3,4-dihydroxybenzaldehyde, and trans-2-hexenal have been immobilized on chitosan films. Figure 4 shows the Schiff base or imine bond reaction between the amino group of chitosan with aldehyde molecules with antimicrobial activity, with which it has been already reported that active molecules were kept stable under neutral pH conditions and their release was activated under acidic pH conditions (Figure 4). The acidic conditions favored the cleavage of the imine bond, leading to the release of the aldehyde. The distribution of the imine bonds all along the film allowed for a fast release until the maximum was achieved, and then over time, a slow decrease in the release rate was observed. Post-modification of the films via imine bonds was found to be a great way to immobilize aldehyde in order to accomplish a fast release under acidic conditions with a more stable phase until the maxim is achieved. This is because the post-modification of the films leads to a surface modification without the need for a complete overture of the polymer.

**Figure 5:**
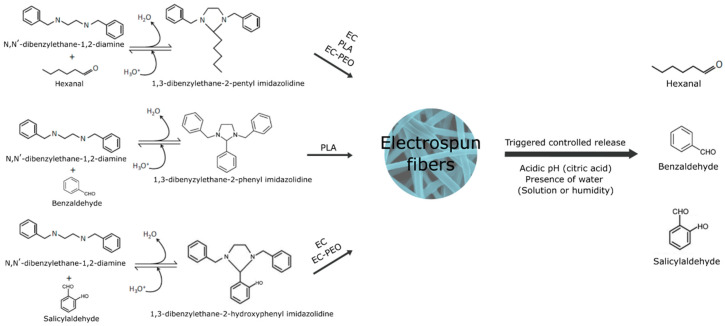
Formation of imidazolidine precursors, their encapsulation as fibers with ethylcellulose-poly(ethylene oxide) (EC-PEO), ethylcellulose (EC), and poly(lactic) acid (PLA) by electrospinning, and the release of benzaldehyde, hexanal, and salicylaldehyde under acidic pH conditions.

Other imine structures are based on the creation of particles or precursors by anchoring aldehydes and, subsequently, incorporating them into other polymeric structures. Thus, Hou et al. [127] generated chitosan particles modified with cinnamaldehyde that were introduced into a copolymerized cellulose and polyvinyl alcohol aerogel to form pH-responsive porous aerogels for food preservation. Other studies employed PEI, and its derivatives such as N,N’-dibenzylethane-1,2-diamine, as a polymeric matrix for the generation of stimuli-responsive precursors with imine bonds [120,121,122]. These works reported the modification of N,N’-dibenzylethane-1,2-diamine through reversible reactions with benzaldehyde, hexanal, and salicylaldehyde to create imidazolidine precursors (imines) in ethanolic solutions. These reactions and their incorporation into different materials are summarized in Figure 5. These imidazolidines were encapsulated in PLA, EC, and EC-PEO fibers via electrospinning, and their release was triggered by the addition of a citric acid solution. The action of an acidic pH triggered a rapid release of the aldehyde due to the imidazolidine present on the fiber surface and a sustained controlled release over time of the aldehyde due to the imidazolidine inside the fibers, requiring the fiber to swell in order to cleave the internal imine bonds. pH- and thermo-responsive imine chitosan hydrogels have been applied in the drug delivery field for the transport and release of antitumoral compounds like cisplatin, DOX, and DTX under acidic physiological conditions [128,129]. Although hydrogels have been widely used to create new structures for the design of biodegradable packaging and the release of bioactive compounds [130], imine-based hydrogels have scarcely been explored in this area, despite showing an improvement in the hydrogel’s mechanical properties when using Schiff bonds for the conformation of the material [131]. 

Other strategies to design stimuli-responsive structures come in the form of micelles [132]. For example, imine bonds have been used in the design of pH-responsive amphiphilic micelles for the stabilization and release of aliphatic hydrophobic aldehydes, such as hexylcinnamic aldehyde, citral, and dodecanal, and aromatic hydrophilic aldehydes such as vanillin, for fragrance applications. 

Despite the fact that different strategies have been developed using stimuli-responsive imine materials for antimicrobial volatile release immobilization, few works have employed this strategy for active food-packaging systems [46,101,102].

### 3.4. Stimuli-Responsive Release of Volatile Compounds in Active Food Packaging

As mentioned above, this novel technology to click and de-click antimicrobial volatiles offers unprecedented opportunities for innovation in material science in many different fields. In this context, considering the potential of these reversible covalent bonds in the stabilization/controlled release of volatiles, some studies in the literature have focused on developing stimulus response systems based on these bonds for their further applications as active food packaging. Their application has been primarily tested through in vitro tests, and examples of their application in food packaging are scarce. Thus, Zaitoon et al. [133] used imidazolidines (as a precursor for salicylaldehyde stabilization through reaction with PEI) encapsulated in PLA/EC-PEO electrospun fibers. To promote the hydrolysis of the imine bond, fibers with citric acid were also developed, which, in contact with a highly-humidity environment, solubilized the acid and hydrolyzed the imine bond, showing effectiveness against *E. coli*. Other studies have explored the potential of imine bonds created on a chitosan film surface with benzaldehyde, citral, hydrocinnamaldehyde, cinnamaldehyde, and citronellal, evaluating the responsive materials against common fruit and vegetable spoilage and pathogenic fungi such as *Penicillium expansum* (*P. expansum*) and *B. cinerea.* These materials showed a higher efficacy when they were subjected to acidic buffer solutions [45,57]. 

Stimuli-responsive materials based on reversible covalent chemistry, tested on food matrices, and incorporated into designs of food packaging have been reviewed, and the main findings on their anchoring/release of volatile compounds are summarized in Table 3. As can be observed, all materials developed for active food packaging are based on imine bonds, where aldehydes with high antimicrobial activity have been covalently anchored. Moreover, the main polymer used to anchor these molecules is chitosan.

The incorporation of reversible covalent bonds in the design of antimicrobial active packaging appears as a novel technology, as it allows the stabilization of covalently anchored volatile antimicrobial compounds, facilitates their handling, and allows their release to be controlled when necessary. As a proof of this concept, Higueras et al. [102] employed chitosan films with grafted cinnamaldehyde via covalent imine bonds to inhibit *L. monocytogenes* in milk. For this, the modified films were placed in contact with milk and subjected to several heat pre-treatments. The release of the cinnamaldehyde became higher with increasing temperatures of the pre-treatment, showing a greater antimicrobial efficacy at 95 °C for 10 min than at 65 °C for 35 min. However, acid stimuli were not considered as a trigger of volatile release. Another study applied chitosan films modified with salicylaldehyde through imine bonds in refrigerated liquid food, in a slightly acidic fruit juice. The acidity of the juice promoted imine hydrolysis and subsequently the antimicrobial release, inhibiting *E. coli* growth in the juice [51].

**Figure 6:**
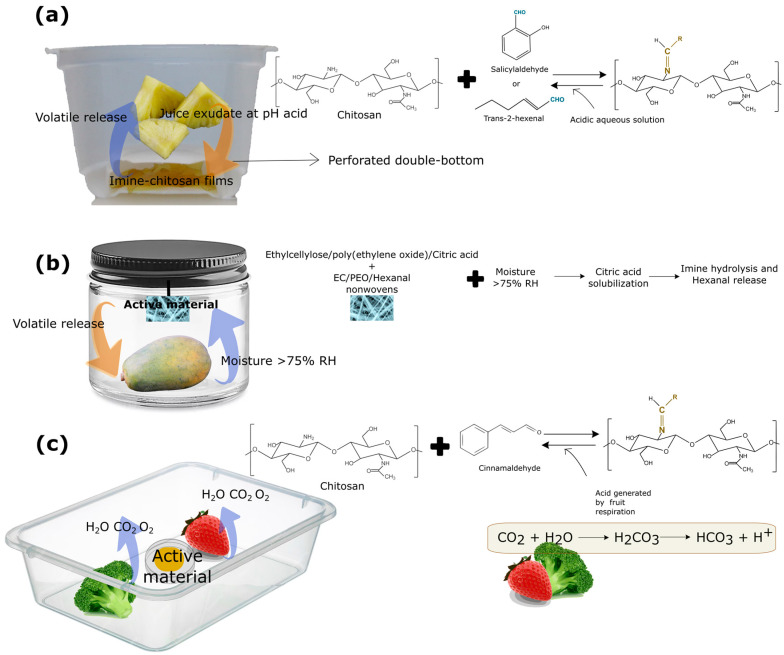
Different designs for responsive polymer incorporation based on reversible covalent bonds of antimicrobial compounds. (**a**) Double-bottom packaging in which imine–chitosan films were placed and triggered by pineapple juice exudate. (**b**) A jar containing papaya and an active non-woven material, wherein aldehyde release was triggered by the generated moisture. (**c**) Release of antimicrobial aldehyde triggered by the high-rate respiration of fruits.

However, these applications required direct contact with the food, since the liquid food medium favored the hydrolysis of the imine bonds. To avoid this direct contact with the food and promote the release in the headspace, different strategies have been developed. For instance, Heras-Mozos et al. [45] developed a perforated double-bottom container of active films for blackberries and fresh-cut pineapple, while avoiding direct contact with the food (Figure 6a). Imine chitosan films modified with trans-2-hexenal and the trigger solution were added at the bottom of the tray and the blackberries were immediately packaged. The hydrolysis of the imine bond due to the change in pH resulted in the aldehyde released into the headspace, exerting its antimicrobial action. In this manner, better handling of volatile compounds was achieved and the shelf life of the blackberries was greatly extended from 3 to 12 days, inhibiting fungal growth on the fruit surface [45]. 

In order to extend the application of this delivery system, this double-bottom tray coupled with imine–chitosan films was employed to packaged fresh-cut pineapple (Figure 6a), so that the juice exuded by the pineapple during storage promoted the imine hydrolysis [46]. The imine–chitosan films subjected to the juice exudate improved the quality of the fresh-cut fruits and reduced their natural microbial load via aldehyde release in the headspace of the packaging. The pineapple’s packaging system, unlike the blackberries’ packaging, allowed for a gradual release of the aldehyde during storage, as the exudate produced by the pineapple promoted the hydrolysis of the bond, without the addition of an external acid aqueous solution. 

Another study increased the shelf life of papaya through the release of hexanal. In this work, the volatile was anchored to a precursor N,N’-dibenzylethylethane-1,2-diamine to form a stable imidazolidine, which was then incorporated in electrospun ethylcellulose/poly(ethylene oxide) fibers with citric acid. This material was placed in a jar with a papaya following the scheme shown in Figure 6b. Upon contact with the moisture generated by the product during storage, the citric acid incorporated in the fibers was solubilized, resulting in the hydrolysis of the imine bond and, subsequently, the release of hexanal, delaying product ripening for 4 days [134]. In addition, Liu et al. [135] demonstrated the high potential of chitosan modified particles with cinnamaldehyde to extend the shelf life of broccoli and strawberries. The release was triggered by the high transpiration rate of these fruits and vegetables, as shown in Figure 6c. In this case, no acidic stimulus was incorporated externally, and the authors reported that the release was promoted by the resulting package headspace from the high-rate respiration of the food together with the generated moisture. 

In general, the application of reversible covalent bonds in active food packaging presents a high potential. Different strategies for the release of the volatile molecule, which is anchored to the polymeric material, have been presented. Further studies are needed on their synthesis, but also on their technological application in the packaging field, as well as on different methods of applying external stimuli to promote the hydrolysis of these bonds.

In this context, on the basis of the reported bibliography, a new packaging concept could be developed, which may be of interest for the application of active materials based on the reversible anchoring of volatiles to create so-called responsive packaging. However, the use of stimuli-responsive materials for food packaging has so far focused on the design of smart packaging [24], and their application in antimicrobial packaging is limited, as mentioned above. Clearly, the application of such systems can be of interest for active food packaging as it ensures a release of the compound on demand, while in the absence of stimuli, it remains stable in the material. 

**Figure 7:**
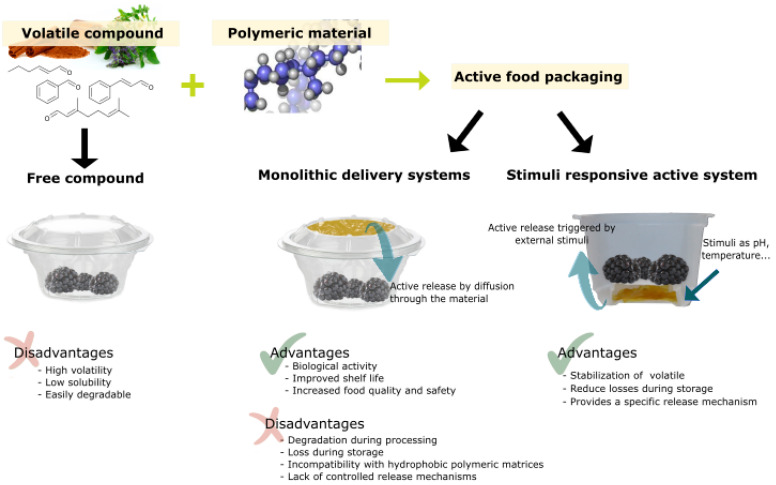
Volatile compound application strategies for active food packaging, showing main advantages and disadvantages.

Figure 7 shows an outline of the strategies for and the advantages and disadvantages of the application of stimuli-responsive antimicrobial polymers in the design of active food packaging. 

Compared to other types of active packaging, a stimuli-responsive active system is only triggered in response to a stimulus, which can come from the food itself. Changes in pH, humidity, or bacteria spoilage occurring during the storage of food could trigger the release of active compounds as a corrective measure in order to maintain the food quality and security. Therefore, the active compound release only occurs when it is needed, in order to exert a corrective response to food deterioration.

## 4. Conclusions

In the present review, we provide an overview of the incorporation of antimicrobial volatiles in polymer matrices through reversible covalent bond reactions for the development of active food packaging. The two main objectives of this methodology are as follows: (a) the stabilization of bioactives in polymer materials, avoiding an uncontrolled release based on molecular diffusion and evaporation and avoiding a loss of the active molecule during storage; (b) the inclusion of a release mechanism to trigger the release of the bioactive when needed. 

The different methods discussed in this paper for purposely incorporating volatile compounds into polymeric matrices attempt to stabilize the volatiles in order to prevent their degradation and loss during processing and storage, as well as to generate suitable controlled-release kinetics. The effectiveness of the release depends not only on the antimicrobial activity of the added volatile compound, but also on its release rate, its volatility, and its compatibility with the polymer and the food product. The main challenges associated with active food-packaging materials based on volatile molecules are the processing methods, which often include high temperatures or pressure, leading to significant loss or degradation of the compound. Furthermore, stabilizing the volatile compound during storage is also a challenge, and its retention in the polymer relies on the method of incorporation and the physicochemical properties of both the active compound and the polymer. Emerging encapsulation and nanotechnology methodologies enable higher retention and lower volatile losses during processing and storage, while at the same time allowing for better control of the release. The choice of method depends on factors such as the specific antimicrobial agent, the packaging material, and the desired release kinetic characteristics. The release of volatiles from most reported antimicrobial active food packaging is based on the diffusion of the molecule through the polymeric material. However, these materials lack a specific controlled-release mechanism. 

On the contrary, stimuli-responsive polymers developed to anchor covalently bonded antimicrobial molecules, the release of which can be triggered by various external stimuli, represent a promising tool, since these smart polymers include specific release mechanisms. Materials based on reversible covalent bonds enable a high stability of the volatile compound during preparation and storage, and its release when needed. In the absence of external stimuli, the volatile remains covalently anchored to the polymer; however, when the material is exposed to certain stimuli such as a change in pH, the bond is hydrolyzed, releasing the antimicrobial compound into the food-surrounding media. Thus, changes in pH, humidity, or bacteria spoilage occurring during the storage of food could trigger the release of active compounds as a corrective measure for maintaining food quality and security. Although these materials have been extensively studied in other areas, their application in active food packaging is scarce. 

This area of study needs further investigation, especially to determinate the factors that trigger the hydrolysis of reversible covalent bonds and their intensity, in order to achieve the optimum release of active compounds for inhibiting or retarding microbial growth in real food products. Due to the complexity of food matrices, most investigations to date have used food simulants and in vitro tests to evaluate the release of active compounds and their antimicrobial effectivity. The application of responsive active food packaging has shown great potential in ensuring food quality and security, extending the shelf life of fresh vegetables, fruits, and meat. However, the technological achievements in this field are limited. Since the design of active food packaging based on stimuli-responsive materials has been scarcely explored, very few studies have developed packaging using these smart materials, but the preliminary studies exhibit considerable potential. In general, further testing on food matrices and full designs of food packages for incorporating these delivery systems should be researched.

## Figures and Tables

**Table 1:** Selected active food packaging containing volatile compounds and its application in real food matrices.

Volatile Compounds	Chemical Class	Method of Incorporation	Food Matrix Application	Bioactive Effect	Reference
Eugenol	Monoterpene	Encapsulation; nanoparticles	Chicken	Reduction of 2 log CFU *Staphylococcus aureus*/g after 5 d.	[34]
		Electrospinning	Strawberries	Reduction in the natural microbial load of the fruit.	[35]
Thymol	Monoterpene	Electrospinning; encapsulation; *β*-cyclodextrins	Meat	Reduction in total bacterial count from 97 × 10^7^ to 11 × 10^6^ after 5 d.	[36]
		Encapsulation; nanoemulsions	Ground beef	Inhibition of total mesophilic bacteria, coliforms, total molds and yeasts, *Staphylococcus* spp., and lactic acid bacteria around 2 log CFU/g after 6 d.	[37]
		Encapsulation; 2-hydroxypropyl-β-cyclodextrins	Tomatoes	Inhibition of *Botrytis cinerea* growth of 66%.	[38]
Thymol and Carvacrol	Monoterpene	Extrusion	Strawberries	*Botrytis cinerea* inhibition.	[39]
Linalool	Monoterpene	Used directly	Fresh chicken breast	*Listeria monocytogenes* inhibition.	[40]
d-Limonene	Monoterpene	Encapsulation; liposomes	Blueberries	Total yeast and mold inhibition.	[41]
Trans-2-hexenal	Aldehyde	Encapsulation; cyclodextrins	Pears	32% reduction in the incidence of *Alternaria alternate* black rot.	[42]
		Used directly	Kiwifruit	*Actinidia chinensis* inhibition.	[43]
		Used directly	Apples	Inhibition of *Penicillium expansum* between 50 and 98% after 24 h.	[44]
		Immobilization; reversible covalent bonds	Blackberries	Inhibition of *Penicillium expansum* and *Botrytis cinerea* after 9 d.	[45]
		Immobilization; reversible covalent bonds	Cut pineapple	Reduction in molds and yeasts by 1.5 log CFU/g after 9 d.	[46]
Cinnamaldehyde	Aldehyde	Encapsulation; nanoemulsions	Mushrooms	*Pseudomonas* reduction of around 2 log CFU/g after 16 d.	[47]
		Encapsulation; nanoparticles	Rainbow trout fillets	Reductions in total viable count by 1.5 log CFU/g and in Gram-negative psychotropic bacteria by 1 and 0.5 log CFU/g after 12 d.	[48]
Citral	Aldehyde	Coating	Salad	Reduction in Enterobacteriaceae, yeasts, and molds by around 2 log.	[49]
Citral and cinnamaldehyde	Aldehyde	Encapsulation; cyclodextrins	Beef	Reduction in total viable counts.	[50]
Salicylaldehyde	Aldehyde	Immobilization; reversible covalent bonds	Cut pineapple	Reduction in molds and yeasts around 2 log CFU/g after 9 d.	[46]
			Orange and carrot juice	2.9 log inhibition of *Escherichia coli* after 6 d.	[51]
Vanillin	Aldehyde	Used directly	Cut melon	1.5 log CFU/g reduction in mesophilic bacteria after 10 d.	[52]

**Table 2:** Reversible covalent bonds used for the capture and release of volatile or active compounds.

Bond	Reaction	Stimuli Needed for Forward/Reverse Reaction	Application	References
Diels–Alder	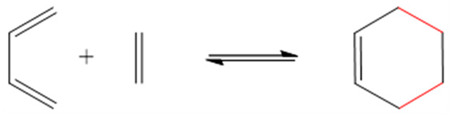	Low temperature/high temperature	Self-healing materials, biomedicine	[87,88,89]
Disulfide	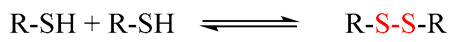	Oxidative or basic conditions/reductive conditions, increase in temperature	Biomedicine	[90,91]
Acetal	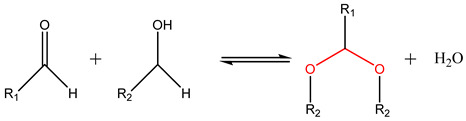	Acidic catalyst/acidic pH	Biomedicine	[90,92]
Acylhydrazone	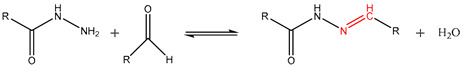	Room temperature, neutral pH/acidic pH, increase in temperature	Biomedicine	[93,94]
Oxime	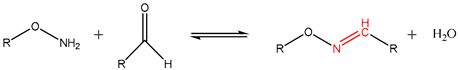	Neutral pH/acidic pH, UV radiation	Self-healing materials, agriculture	[95,96,97]
Imine	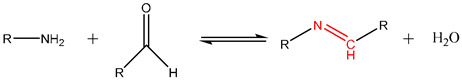	Neutral pH/acidic pH	Biomedicine, pharmaceutics, agriculture, cosmetics, food packaging	[20,46,84,98,99,100,101,102]

Note: The new reversible covalent bonds are shown in red.

**Table 3:** Antimicrobial food-packaging systems based on reversible covalent bonds of volatile compounds.

Volatile	Polymeric Material	Covalent Bond	Responsiveness Stimuli	Food Matrix	Reference
Trans-2-hexenal Salicylaldehyde	Chitosan	Imine	Acidic solution provided by food exudate	Pineapple	[46]
Trans-2-hexenal	Chitosan	Imine	Acid solution added externally	Blackberries	[45]
Vanillin	Chitosan	Imine	Not specified	Raspberries	[101]
Hexanal	Imidazolidine precursor incorporated in fiber of ethylcellulose/poly(ethylene oxide)	Imine	Moisture generated by fruit triggered the acid citric solubilization to promote imine bond	Papaya	[134]
Cinnamaldehyde	Chitosan complex embedded in cellulose nanofibers (CNFs) and polyvinyl alcohol aerogels	Imine	Juice exuded from fresh pork	Fresh pork	[127]
Cinnamaldehyde	Chitosan	Imine	Fruit respiration to form CO_2_ + H_2_O → HCO_3_ + H^+^	Broccoli and strawberries	[135]
Salicylaldehyde	Chitosan	Imine	Acid present in liquid food	Fruit/vegetable juice	[51]
Cinnamaldehyde	Chitosan	Imine	Temperature and liquid food	Milk	[102]

## Data Availability

No new data were created or analyzed in this study. Data sharing is not applicable to this article.
